# Identity Salience and Field‐Course Engagement: From Deficits to Assets

**DOI:** 10.1002/ece3.73230

**Published:** 2026-03-15

**Authors:** Alyssa N. Olson, Vigdis Vandvik, Sehoya Cotner, A. Kelly Lane

**Affiliations:** ^1^ School of Biological Sciences University of Nebraska‐Lincoln Lincoln Nebraska USA; ^2^ Biological Sciences University of Bergen Bergen Norway; ^3^ Biology Teaching and Learning University of Minnesota Minneapolis Minnesota USA; ^4^ STEM Education Research Center University of Bergen Bergen Norway

**Keywords:** asset‐based pedagogy, concealable stigmatized identities, field courses, hidden identities, identity salience, inclusion, inclusive pedagogy

## Abstract

In contemporary higher education, teachers and administrators must make choices about how best to allocate resources for maximum benefit. In many STEM fields, this has led to closer scrutiny of many costly offerings, such as laboratory and field experiences. In response, several investigators have highlighted the many educational benefits these high‐impact practices provide, while also shedding light on concerns related to diversity, equity and inclusion. Field courses are especially notable for disproportionately engaging students from high socioeconomic backgrounds, racial and ethnic majorities, and—in some disciplines—men. To address inequities in STEM, field courses should therefore be investigated to better understand students who are underrepresented in the disciplines—whether based on their nationality, language proficiency, socioeconomic background, race or ethnicity, their gender identity or sexuality, etc. Here we report on findings from a series of interviews in which students in an international, graduate‐level field course discussed which of their identities, either visible or hidden, were most *salient* in the field‐course context. Respondents reflected on how these identities served as either promoters or barriers to engagement in the course, and shared course interactions that intersected with these identities in either positive or negative ways. Our analysis of these interviews allowed us to identify several key themes, among them the idea that the interviews themselves are a type of pedagogical intervention that instructors could adopt to promote inclusion. By identifying how student identities interact with course practices to impact engagement, we can begin to outline best pedagogical practices for field courses. Thus, we can ensure investments in these experiences benefit *all* students and help to make STEM diverse, equitable, and inclusive.

## Graphical abstract

To address inequities in STEM, field courses should therefore be investigated to better understand students who are underrepresented in the disciplines—whether based on their nationality, socioeconomic background, race or ethnicity, their gender identity or sexuality, etc. Here we report on findings from a series of interviews in which students in an international, graduate‐level field course discussed which of their identities, either visible or hidden, were most salient in the field‐course context. Our analysis of these interviews allowed us to identify several key themes, among them the idea that the interviews themselves were a type of pedagogical intervention that instructors could adopt to promote inclusion.

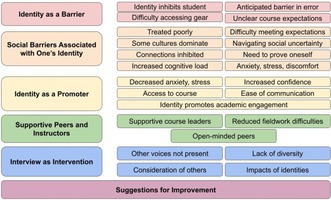

## 1 Background

Several investigators have recently highlighted the educational benefits of field courses in STEM higher education (Shinbrot et al. 2022; Zavaleta et al. 2020). This research has partly been motivated by concerns over the high costs of field courses, and hence the risk that institutions might cut back on such courses for financial reasons. For this work, we define “field courses” as formal educational offerings (typically credit‐bearing and embedded in a curriculum) that involve multi‐day off‐campus living and working in a “field” setting. Benefits of these experiences can relate to fulfilling course learning objectives by connecting theory to practice (Geange et al. 2021; Durrant and Hartman 2015; Hoyer and Hastie 2023); or increasing student motivation (Fägerstam 2014; Treibergs et al. 2022), self‐efficacy (Beltran et al. 2020), and sense of belonging in a specific area of study (Shaulskiy et al. 2022); or helping develop transferable skills such as communication, planning, and flexibility (Peasland et al. 2019). Further, international field courses can promote the development of cross‐cultural communication skills (Brendel et al. 2016; Arboleya and González‐Díaz 2021). Collectively, these benefits can translate into palpable professional gains. A recent report on 50 years of a single graduate‐level field course at Cornell University (Arcila Hernández et al. 2023) found that field course engagement was associated with higher academic success, with course participants becoming faculty at a higher rate than their peers. For these and other reasons, field courses have been cited as an example of a *high‐impact educational practice*, alongside other types of authentic engagement such as student research experiences and internships (Arcila Hernández et al. 2024; Kuh 2008; Kuh and O'Donnell 2013).

However, there are significant barriers to educators' implementation of and students' engagement in field courses. In contrast to campus‐based educational practices, field courses incur significant financial, practical, temporal, and personal costs. Students and instructors must be transported to a field site; they need on‐site food and housing, and there may be additional costs associated with travel, insurance, personal field equipment and safety gear, etc. (Giles et al. 2020). Travel to field sites can take additional time, and this can have a personal cost in terms of time away from other commitments–work, family, and other courses (Pierszalowski et al. 2021). And the physical demands of some field courses can be significant (Giles et al. 2020).

The intense, around‐the‐clock nature of living and working together during a field course can create a positive experience that promotes a sense of belonging and engagement within a discipline (Strayhorn 2018; Shaulskiy et al. 2022; Esparza and Smith 2023). However, this sense of belonging is likely not distributed equally among all students, but varies based on features of a student's identities (Carlone and Johnson 2007). For example, prior work has indicated that women can be victims of sexual harassment at field stations (Clancy et al. 2014), a major problem that has been found to hamper efforts to diversify fieldwork‐centric disciplines such as the geosciences (Marín‐Spiotta et al. 2020; Giles et al. 2020) and ocean sciences (Maia et al. 2024). Despite evidence suggesting field‐course experiences may lower demographic achievement gaps (Beltran et al. 2020), data collected in the United States show that people of color are notoriously underrepresented at field stations, which may be due to several factors, including economic limitations (Jensen et al. 2021) and persistent racism (Marín‐Spiotta et al. 2020). Furthermore, many field stations have lagged behind in providing basic accommodations for people with disabilities (Rudzki and Kohl 2023). While we acknowledge that there may be physical requirements for completing some or all course activities in the field, recent work has highlighted how physical fitness can be over‐emphasized in field‐course settings, leading some students to feel that one must be in peak physical condition to engage in, for example, geosciences fieldwork (Giles et al. 2020).

Several recent reports have indicated the need to go beyond gender, race, and physical ability in investigations of how identities can contribute to a sense of belonging in higher education, and in STEM in general, encouraging researchers to also consider other and often hidden aspects of students' identities (which may be considered “concealable stigmatized identities,” CSIs; Quinn 2006; Henning et al. 2019). For example, students with minoritized sexual identities (e.g., gay, lesbian, bisexual) or gender identities (e.g., transgender or nonbinary) are less likely to remain in STEM disciplines than their peers (Hughes 2018), with reports showing that they can face multiple types of harm and discrimination in STEM environments (e.g., Casper et al. 2022). Engagement in STEM courses has also been shown to be affected by psychological characteristics, such as having attention‐deficit hyperactivity disorder (ADHD; Wang et al. 2024; Hotez et al. 2022), depression (Cooper et al. 2020), or anxiety (Cooper et al. 2018; Downing et al. 2020). Finally, sociocultural characteristics, such as being a first‐generation college student (D'Amico and Dika 2013; Núñez et al. 2020), or coming from a low socioeconomic‐status background (Niu 2017) can also impact engagement in STEM courses. These identities may be more important in some settings than in others. For example, lesbian, gay, bisexual, transgender, or queer (LGBTQ) students may feel more “exposed” during some course activities, such as the informal interactions that can occur in active‐learning settings (Cooper and Brownell 2016). Transgender, gender nonconforming, intersex, and nonbinary students may find certain science topics like discussing sexual dimorphism in biology courses to result in challenging interactions with their instructors and peers depending on how the content is presented (Casper et al. 2022). Importantly, the listed negative outcomes for those with CSIs could be further exacerbated by the field course environment, as it is often isolated and the distinction between professional and social activities is less clear. Students attending field courses often have communal living spaces, shared meals, and shared work and social activities, leading to more opportunities for the aforementioned negative student outcomes to be realized (Hall et al. 2004).

Furthermore, while much of the previous work on CSIs emphasizes specific identities as serving as potential *barriers* to engagement, we realize that a diverse range of lived experiences, perspectives, and wells of knowledge can be viewed as assets that *promote* engagement. Asset‐Based Pedagogy (ABP) is a pedagogical approach that aims to eliminate deficit‐based thinking, whereby “difference” is a problem, and supplant that with asset‐based approaches (López 2024). With deficit‐based approaches, well‐meaning instructors may seek to “remedy” problems that are features of a student's identity or background; here, the emphasis is on what a student *lacks* (Dudley‐Marling 2015), how their cultural background has *failed* them, or how there are *gaps* between students based on these features (e.g., Black versus White students, male versus female). Conversely, ABP sees difference as a strength, and seeks to provide opportunities for students to draw from their full range of existing cognitive resources—whether these are the result of formal schooling, life experiences, identities, or their extended networks. We note that field courses, where there is typically a focus on practical and problem‐solving skills and learning activities outside what is considered the traditionally “academic” realm, could offer opportunities to promote engagement across diverse student identities. However, an asset‐based perspective is lacking in the current research on field courses.

While there have been calls to consider hidden identities in respect to field courses (e.g., socioeconomic background, gender, and sexual orientation; Lundin and Bombaci 2023; Nash et al. 2019), research into this topic lags behind the need to address these students' experiences (Morales et al. 2020). Additionally, the authors are aware of no studies that identify promoters associated with CSIs or other minoritized identities in field courses. The lack of research on individuals with CSIs in field work is a notable shortcoming of the current literature, given that student identities are likely more *salient* (i.e., prominent, important, active in the students' own thoughts) in the confined and intense environment of a field course–where students may be together 24 h a day, with nontypical academic tasks such as practically oriented and outdoor work, sometimes under extreme or unusual conditions, and with close interactions and interdependence within a limited group of people, many of whom were strangers prior to the course. These settings offer challenges, but also opportunities for students with a variety of experiences and knowledge to contribute meaningfully in a group setting.

### 1.1 Research Questions

The present study was motivated by this lack of information about how hidden identities impact engagement in field courses. We approached the topic broadly, by considering which of many possible identities were most *salient* to students participating in an international, graduate‐level field course. We define *salient identity* as the elements of one's identity that they perceive to impact their experiences within the context of this field course (Thompson et al. 2020). In this work, we specifically sought to understand, through analysis of interviews, student perceptions of identity salience in field courses and how instructional choices could minimize identity‐related barriers and maximize identity‐related promoters to engagement. It is important to note that the aim of these interviews was not to assess the specific course (which is often done through different mechanisms, including pre‐ and postcourse questionnaires), but rather to engage with students' experiences with field courses generally in light of their salient identities, using this course as a context in which to have those conversations. Our initial research questions were:

1. *Which identities were salient to students during this international, graduate‐level field course?*
2. *Did identity salience serve to promote or serve as barriers to student engagement in this course? Specifically, in what ways did identity salience promote or serve as a barrier to student engagement in this course?*

After our initial qualitative analysis, a third research question was evident:

3 *How Can We Use Insight From These Interviews to Design Courses That Promote Inclusion for Participants From a Range of Backgrounds and With Diverse Identities?*

As a final outcome, our hope was to use student narratives to make recommendations from an “asset‐based,” rather than a “deficit‐based,” framework of identity salience in field courses, which we include in our discussion.

## 2 Methods

### 2.1 Course Context

The focal course for this work (“Plant Functional Traits”) is an international, graduate‐level ecology course that was in its sixth iteration during our interviews (July 2022). The aim of this research‐based course is to “offer hands‐on training in different applications of plant functional traits ecology within a real‐life field research project setting,” thereby “providing students with essential background knowledge and the practical field, lab, and computational skills needed for conducting their own research within trait‐based ecology.” Doctoral and masters students are recruited from around the world, based on open advertisement of the course and promotion through diverse academic networks. Potential students are selected based on the students' motivation letters in which they are instructed to explain how and why this course would promote their academic development and fulfill their training needs. Selection is a “blind” process where identities (gender, nationality, institution) are hidden to assessors. For each course, students from the region where the course is taking place are prioritized, if necessary (aiming for at least 50% regional students), to reduce carbon footprint and promote regional networking. Course fees vary depending on the GDP of the country of the students' home institution, and can be fully waived upon application, if the students' circumstances suggest the fee is a barrier to their participation.

Students meet in remote Zoom sessions several times prior to the in‐person course to discuss relevant literature and to clarify the course research projects, philosophy, and expectations. These sessions are recorded and available on the course home page for review. On site, they work in five teams of 5–7 students with 1–2 instructors per team who plan, conduct, and report on research projects such as plant traits, leaf and ecosystem carbon fluxes, and remote sensing along gradients of climate and land‐use. The final course deliverable is a published data paper, co‐produced by the students and instructors and any local collaborators, reporting on the study system and data (see Vandvik et al. 2025). After the course, students are encouraged to collaboratively develop and publish research papers based on the course projects (e.g., Henn et al. 2018; Thomson et al. 2021; Roos et al. 2025).

For this offering, the course involved 29 students from 17 nationalities, affiliated with institutions in 13 countries, about half from within the course region Europe (48%), with the rest distributed based on availability of applicants from the Americas (41%), Asia (3%), Africa (3%) and Oceania (3%). These students and 12 instructors (50% Europe, 50% Americas) spent 2 weeks in Aurland, Western Norway. Students lived, did lab work, and had communal meals at a local organic‐farming school, and took day‐long and overnight excursions to nearby field sites to conduct in situ experiments, collect plants, and make measurements. The course was conducted in English. Prior iterations of this course (in Asia, South America, Europe, and Africa) have been documented in other reports (e.g., Geange et al. 2021; Patrick et al. 2020). See also the course web page for course structure, content, regional attendance in the various courses, and course philosophy (https://plantfunctionaltraitscourses.w.uib.no/).

### 2.2 Positionality and Reflexivity

The sole coder and interviewer in this study (ANO) held a position as both insider and outsider when conducting the interviews and analysis (e.g., Dwyer and Buckle 2009; Mulholland 2007; Parson 2019). Due to the travel required for the course, ANO was a participant in the course, traveling and completing nearly all course activities including socialization opportunities with the students. At the time of the course, she was a research intern who had not yet begun graduate school. The students were informed that ANO was conducting this research in collaboration with two of the instructors of the course (including SC and VV). As a result of her participation in course activities, she developed interpersonal relationships with the study participants. This likely impacted both data collection and analysis. Through all stages of the study including study design, data collection, analysis, and writing, one or more team members reflected with ANO on how her identities and course involvement may have impacted data collection, analysis, and interpretation. Several participants expressed that they were comfortable sharing personal anecdotes with the interviewer and participating in the study. For other participants, the relationship between ANO and course leaders (i.e., the professors on the course) could have been a reason to withhold personal views and information during the interview or choose not to participate, ultimately impacting what data we had access to as researchers.

While it is impossible to know how ANOs status as both insider and outsider impacted what data she was able to gather, the authorship team took steps to ensure that multiple perspectives were included in the interpretations presented in the final manuscript. ANO's analysis was overseen by AKL, who was not involved with the course. AKL has a breadth of experience conducting qualitative research, especially with interview data and has a personal epistemology that all statements in qualitative work should be supported by evidence in the form of quotes. AKL also encouraged ANO to hunt for counterexamples and negative cases to claims before they were finalized in the manuscript (Creswell and Miller 2000; Booth et al. 2013). It is notable that both AKL and ANO are American and thereby do not share a cultural identity with many of the course instructors, nor do they share one with many of the participants. This could have limited their perspectives. VV provided the insider perspective both as a long‐term co‐developer of the course and as an academic from the specific field of study and study region. Finally, the writing of the manuscript was led by AKL and SC. SC has experience with the literature around field courses and hidden identities and was a co‐organizer of the course while AKL lent her experience with theory, qualitative analysis, and issues regarding equity. In this way, the manuscript was written by both an insider and an outsider to the course itself, with ANO answering questions about context, identifying quotes, and aiding in interpretation as needed. These steps were taken to enhance the rigor of the findings and analyses.

### 2.3 Data Collection and Participant Recruitment

In the confidential precourse survey, students were asked to report which identities they hold, including age, nationality, educational background, hidden or visible disabilities, religiosity, politics, sexual orientation, gender identity, generation in college, socioeconomic status, ethnicity, native language, or anything else that the student considers to be part of their identity. They also indicated if they had interest in speaking with ANO about their course experience during or after the course, in which case they consented to providing information about their declared identities as part of the research process (see below). Upon seeing the range of identities represented in the course and interest in participating in an interview, SC recruited those interested students by email during the course. As interviews began, additional students expressed interest and thus were interviewed and included in the analysis. In total, ANO interviewed 15 students.

To protect student anonymity, our reporting of identities will not include a specific number of students claiming an identity, specific countries of origin, or other potentially identifying characteristics.

We followed national regulations regarding the use of human subjects (confidentiality, informed consent, ability to withdraw at any time). Our research was notified to the appropriate authorities at the University of Bergen and was registered as R2084: Field courses and student identity salience.

The interviews were semi‐structured (McGrath et al. 2019; Siedlecki 2022) and began with a short introduction to the project. The discussions were anchored around the following questions:

1. Which of your identities, if any, have been most noticeable to you as promoting your engagement in this course?
2. Which of your identities, if any, have been most noticeable to you as barriers to your engagement in this course?
3. How has this identity manifested itself in the course?
4. How does this identity salience impact your experience?

The full interview protocol is shared in the supplemental materials. Interviews lasted between 15 and 60 min and were transcribed by ANO. After transcription, names and nouns were replaced with pseudonyms or other designators to protect participant identities. All quotes presented in this manuscript have been lightly edited for grammar and clarity with any significant word changes or additions indicated by brackets. To protect the confidentiality of participants we do not present quotes with participants' designators or pseudonyms. Though some participants held multiple CSIs or nonconcealable stigmatized identities, they usually described the salience of each identity separately. All quotes, unless otherwise noted, were in reference to salience of a single one of a participant's identities. Additionally, we refrain from describing whether identities were hidden or not, as it was not always clear to the interviewer whether others in the course were aware of a participant's concealable identity. Since there was a relatively small pool of potential participants, we erred on the side of caution in presentation of all data, choosing quotes that functioned as evidentiary while maintaining participant confidentiality.

### 2.4 Qualitative Analysis

For this analysis, we followed the basic tenets of reflexive thematic analysis, which iterates over six core phases: familiarization, generating codes, constructing themes, revising themes, defining themes, and writing the final manuscript (Braun and Clarke 2006; Braun et al. 2018; Saldaña 2016). ANO conducted and transcribed the interviews followed by re‐reading and identifying initial ideas within three of the transcripts. ANO then proceeded to code the data using an inductive approach where the coder does not come in with a preconceived set of codes but rather defines the codes iteratively during data analysis. This coding process was iterative in that it involved consistently returning to previously analyzed interviews to re‐divide and re‐define codes as new ideas were identified in subsequent analyses. During this process, ANO was mentored by AKL in how to proceed through coding; however, ANO defined and applied the codes solo.

After ANO completed coding by reviewing each interview transcript at least once, she met with AKL and SC to construct initial themes. Some of the themes ultimately served as domain summaries—rich descriptions of the range of ideas in a given category as presented by the study's participants—while others did not meet the expectations of a theme as defined in reflexive thematic analysis as a “pattern of shared meaning” (Braun et al. 2014). Initial theme construction involved ANO sharing her perspectives of the overarching ideas present in the data, and then SC and AKL asking questions regarding the codes and their relationship to ANO's ideas. ANO was then tasked with identifying quotes that exemplified each code and also identifying quotes that challenged the core meaning of each theme, if possible. Any evidence that was contrary to a theme was carefully considered and the theme or its presentation was revised to account for all evidence, as far as is possible. This revision stage was iterated multiple times throughout the analysis.

**FIGURE 1:**
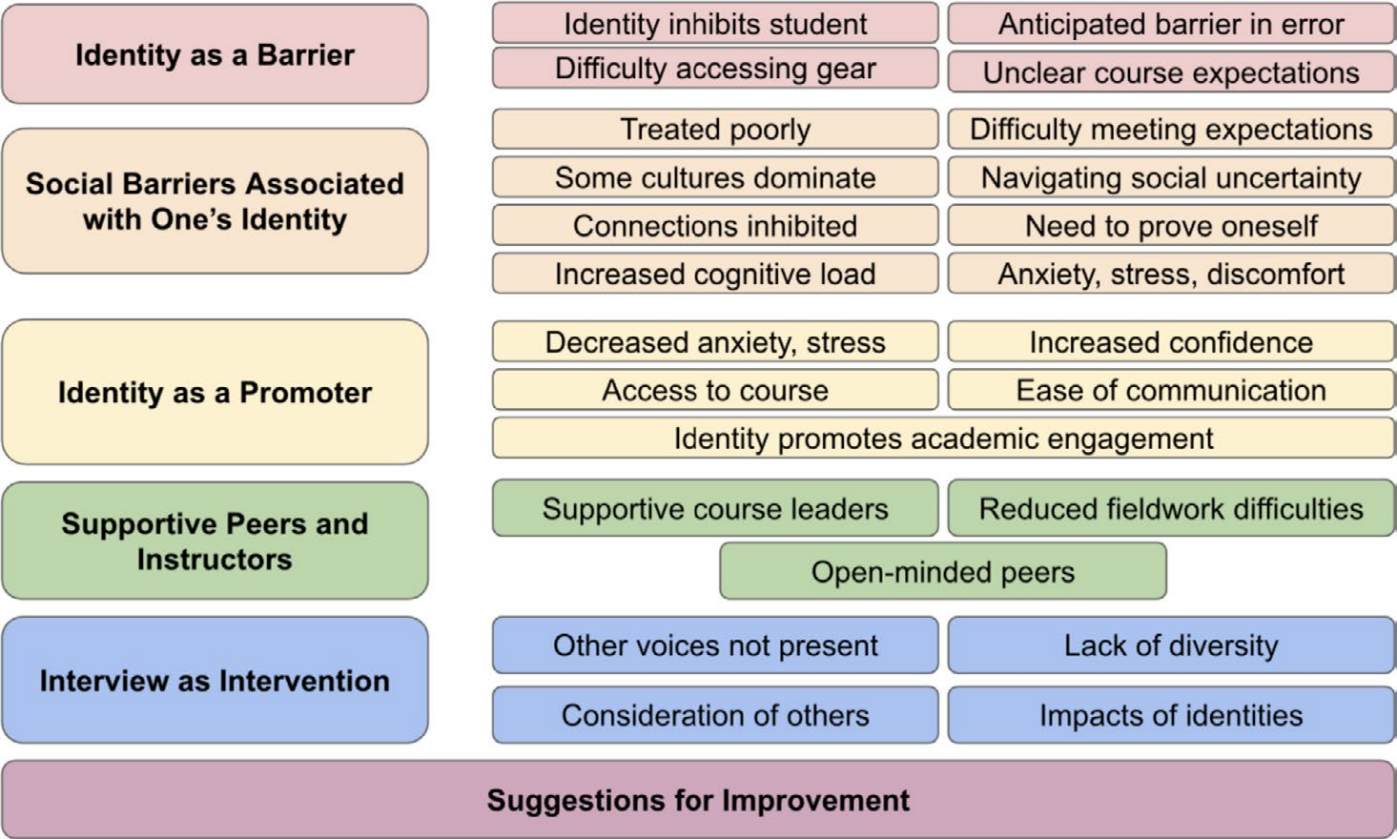
The six themes, and associated categories, that resulted from qualitative analysis.

AKL and SC began constructing the narrative based on these quotes, codes, and themes which ANO then reviewed comparing the presentation of the written themes with the data and the final codebook. We then iterated over the themes once again returning to the data as necessary with VV supplying external critique of the presentation of the themes to make sure they were accessible to an audience that was not able to review the complete transcripts. The codes that were used in identifying evidence for each theme are shown in Figure 1.

## 3 Results

Our 15 interview participants shared a range of salient identities, including some that have been investigated more thoroughly with respect to field‐course engagement (e.g., gender, race, first generation academic, non‐native speaker of the course language) and many that have not (e.g., sexuality, religiosity). Several respondents indicated identity salience related to nationality, cultural background, native language, generation in university (i.e., first or continuing), socioeconomic status (SES), age (i.e., being older or younger than the group at large), educational background (e.g., having little or extensive fieldwork experience), gender (female, male, or gender nonconforming), politics, religion, and ethnicity/race. Fewer noted specific sexual orientations, physical disabilities or abilities, or neurodivergence (often described as someone whose brain works differently especially in regard to processing information and can include conditions such as autism, ADHD, mental health concerns and more). Many participants specifically described identities they held as those of privilege, such as being of certain nationalities or cultural backgrounds, White,^1^ high socioeconomic status, cis‐gendered, able‐bodied, and/or heterosexual.

Our analysis resulted in six core themes that address our research questions and lead to practical suggestions for improvement of field courses. We constructed these themes based on the qualitative coding and literature review. One of these themes–suggestions for improvement–corresponded to a single code in our analysis, but all others drew on multiple codes as evidence of the theme (Figure 1). Below we describe each theme using quotes from the participant interviews as evidence of our claims. All quotes have been lightly edited for clarity (e.g., removing extraneous ums, likes, etc.) and to protect the identities of the participants; however, the underlying meaning of the participants' statements was always maintained. Brackets indicate words that were changed or added for clarity/anonymity and brackets with italics indicate other cues beyond words that a participant used such as laughing.

### 3.1 Identity as a Barrier for Navigating a Field Course Setting

One theme, *Identity as a barrier*, encompassed several categories of input. Several respondents spoke about how their *identity inhibits them academically*, the details of which often varied alongside the identities of the participants. A repeated refrain, however, was that navigating the hidden curriculum of field courses and interacting with those in charge can prove difficult for those with underrepresented or marginalized identities. One participant, who has multiple historically marginalized identities, described their experience this way,

Being a first‐generation student, it gives you this like sense of awkwardness at times because you don't know how certain social hierarchies work in academia and I think that becomes very obvious when, for example, you might be interacting with people who are in senior positions in science, or in academia. Like, say when you're talking to someone like [course leader], right. This is like a person that is famous and you've read their papers since you were in undergrad. And then you don't really know how to interact with someone like that.

Those with less visible identities could also feel anxiety or discomfort while talking to those in charge, concerns that seemed to be due to their own awareness of their identity and the way it impacted their behavior in the course. One participant described having to ask a course leader to repeat themselves multiple times, saying,

My [neurodiversities] slow [my processing speed] down, so I don't understand still, like I'm still processing it. So then it has to be repeated like a third time. Which then gives me anxiety because now I've asked for the same thing to be told to me multiple times. And then it's still processing. And then like on the fourth time I'll finally get it.

For this participant, being aware of how their brain processes information may have helped them identify what support they needed, but it also made them aware of the differences between their behavior and their perception of the behaviors of other students in the course.

While these particular examples are focused on social interactions, other barriers were more practical and specific to a field‐course environment. For example, due to their backgrounds, some participants felt they had more *difficulty accessing appropriate gear*. One participant shared, “If you don't have the financial means to purchase the items that are listed in the packing list, you might have an uncomfortable experience.” Socioeconomic status was only one of many factors that could prevent students from fully engaging in the course activities. Students with physical disabilities or who were older than others also described *struggling to meet the physical expectations* of the course, with one participant sharing,

I do notice that it's a little bit harder for me to hike than I expected. And I feel like “oh, I should be able to keep up.” … And so, you know, nobody is giving me crap for it or anything. It's all totally self‐imposed and everyone's been very kind about it, but there have been moments where I'm like [*laughs*] “oh man, it's a little bit tough. It didn't used to be this tough. That's too bad.” [*laughs*].

In these two prior quotes, the participants are noting practical, visible, and immediate barriers to them being able to fully participate in the course based on their identities or physical limitations.

Participants also noted that they came into the field course with an *apprehension of barriers* that did not come to fruition. One participant had previous experiences with discrimination as a woman in field courses.

I'm always apprehensive when I go to do courses as being a woman, how it's going to be. It has proven not to be an issue in this course, but I have done other courses in different parts of the world where either you're the only woman or you're in a group of women, but then there's like a really competitive feel to it and then it's not very healthy.

Another participant felt like they “had to behave” in a certain way due to their nationality. They were concerned that they would be treated in a certain way, or that others in the course may expect them to behave a particular way, but noted that this concern ended up being a non‐issue for them on this particular course.

### 3.2 Social Barriers Associated With One's Identity

This second theme emphasizes social barriers between peers, specifically those that stem from the expression or suppression of a salient identity. One participant described being *treated poorly based on their identity*, which for them was due to their nonconcealable stigmatized identity.

I think people kind of show very quickly whether they're like on board with being around someone that's not the same [identity] as them. It becomes very, very apparent who is down for it and who's not… sometimes [you] get the sense that others, you know, they make it very clear that they do not want to interact with you, and [*brief pause*] you can't help but wonder why.

In this case, the participant noticed that peers in the program avoided interacting with them due to their identity. They further explained,

I think there were some participants in the course that did look genuinely uncomfortable with my presence… I felt like I was being looked at, like a zoo animal somehow, like not like a person…you would see them introduce themselves to other … people and be like ‘ohhhh,’ all bubbly and nice and then with you it's kind of like ‘oh I don't know how to talk to this [*long pause*].’ That was a strong feeling.

Here, the participant expands upon these feelings of being discounted because of their identity, noting that they did not simply feel ignored but rather felt like they were being treated or viewed as less than human. While only a small number of participants had this type of experience with their peers, instances where participants felt they were being looked at differently or treated poorly were in relation to one of the participant's identities. These instances occurred as early as the first interaction between peers, potentially setting the tone for a student's entire field course experience.

Participants with a variety of identities such as being neurodivergent or a non‐native English speaker also found *difficulty meeting social expectations* of a field course. One participant with a historically marginalized identity discussed the unique social nature of field courses and how that impacted their energy and ability to engage in the course;

I'm with these people for so long with such little reprieve that I can't really escape, you know, because we're all like, staying together in the same building. We work together all day long, and that constant [*pauses*]. So I'm never given a break from, like navigating those feelings and trying to read people. I feel like it's just constantly reading people on what they're thinking.

This constant presence and interaction was exhausting for this participant, but they did note later in the interview this stress diminished over time. Once they learned that their peers were accepting of their identities, their identities became *promoters* to their engagement because they were able to be their full self.

For some participants with CSIs or nonconcealable stigmatized identities the burden of social interaction was about more than sheer abundance; these social interactions also demanded *increased cognitive load* and concerns over others' opinions as they *navigated social uncertainty*. One participant that is a non‐native English speaker described their increased cognitive load this way,

It takes sometimes longer time to define what exactly I want to say and saying it the right way. And then there is also this feeling that sometimes that maybe I say something in a wrong way, just thinking again [*chuckles*], or rethinking what I've said, like whether that was like the right way to choose the words.

For this participant, each social interaction required additional processing time, further compounding the social pressures they felt. Another participant described the burden of navigating their salient identities in interactions with new people,

You kind of have this like hump you have to get over… I was explaining like getting a feeling for the course and what peoples' not only acceptance level of, you know, [my identity], but also … people might know, but do people want to hear about it? Like that's different. It's like figuring out one, is it safe to even just disclose it and people know it. And then: Can I actively live in that identity during the course?

Acceptance of their identity was only part of this participant's concern. Ultimately, they questioned if they could be their authentic selves during the field course. It was one thing for others to be verbally accepting of their identity and another to feel like they could be living that identity and still feel comfortable.

The social aspects of field courses could also alienate students from various cultural backgrounds, especially if they are the only individual with a given cultural background or if a quorum of the students at the course was from a single culture that was different than that of the participant. At this particular field course, participants shared that *certain cultures dominated* the dialog and drove the social interaction between students.

There were several occasions when we were in the group and then I said something and like nobody heard it or I said something and someone else started to talk about something totally different, like on top of me… But I noticed that [the students from this cultural group] also did it to each other and that they didn't seem to mind.

The participant observed that this type of social interaction bothered them, but that it didn't seem to be an issue among students who were from one particular culture. This same participant continued to describe the cultural differences that they noticed saying,

One of the things is the interruption, and that [the students from this cultural group] are just louder, but also that they kind of never stop talking [*laughs*]… it's like they take over… the next person to talk starts talking a bit before the other person is done and if they stopped talking, the last person that was talking would just make sounds, kind of [*laughs*]… So just… the sound space is kind of always occupied [*laughs*].

Having a dissimilar identity with peers can *inhibit connections* with other people in the course. One participant described the dynamic this way,

Maybe those cultural differences between the people in your group are quite big. And instead of mitigating it by having some interactions with some of the other [groups], where maybe your cultural differences are more in line… then you don't have that because you're sort of just in your little group.

To add context, the field course in this study had students divide into smaller groups to conduct research associated with the course. This participant is describing the challenge of being in a group with varying cultural backgrounds. Due to the structure of the course, it was harder for this participant to connect with students in the course who did share their cultural background.

The combination of these social challenges could leave participants feeling like they *needed to prove themselves* within the course setting, such as this respondent reflecting on having a minority identity: “I feel like you have to like prove yourself a lot more and [*pauses and sighs*] it's almost like you can't be taken seriously in that context somehow.” To this participant, their identity meant that they had to go further to prove their belonging in a program in a way that others with majority identities did not have to do. Another participant saw this need to prove themselves as a challenge,

Like if I work hard enough and am usually right then no one can shut me down for [identity]. Like, I think that it's like this expectation, like I will be the best so that you cannot get rid of me or exclude me.

This participant saw being the best and fighting to prove themselves as a way to indicate to others that they belong at the field course regardless of their identities.

Overall, these social challenges could lead students to compare and contrast themselves with their peers. One participant who had come close to experiencing houselessness described these comparisons as semi‐conscious and constant, “I feel like it always occupies a small percentage of my brain in some ways and it's where I just am always making small comparisons. Some that are like, I felt very accurate, like there are very clearly few people [in Norway] that appear to be house‐less.” Another participant expanded on the idea of comparison saying,

So you're entering into this thing that feels a bit like an ivory tower and I feel like it makes you feel really mixed up inside and have like this internal conflict about the work you're doing and its value. You also feel a sense of being separate again from the group of people that you're working with because you have a sense that quite a few of them are not from a working‐class background and are not first generation. And your expectations and your familiarity with the world of academia is just completely different.

These feelings of being different from their peers led this participant to feel conflicted about being at the course at all,

I felt really guilty for coming to the course, because I was like these courses cost money that my home institution is going to pay for. That feels weird. And…like I used to work [in a service profession] … And then to have someone else [providing those services to me] it's like I feel like that's all these layers of experience that are positive but also a lot of guilt, somehow.

For many of the participants with CSIs, marginalized, or underrepresented identities, the abundance of social interaction that accompanies field courses presents challenges that may not be as apparent in traditional course settings. In a field course, students live, eat, and work with each other, often for several weeks, resulting in additional social barriers to course engagement.

### 3.3 Identity as a Promoter of Engagement

Some participants' comments suggested an awareness that their salient identities could serve as assets in this environment. Many participants referred to these identity‐based assets as “privileges.” In some cases, an identity could help students to be *more confident* in themselves. The mediating factor concerned the individual's comfort with their identities rather than the identity itself. One participant noted that their life experiences have led them to be more comfortable with their hidden identities, sharing:

I think when you're [of a certain age] you're much more sure of who you are. And so I feel a lot more confident when I say something…I used to be a lot more insecure about my intelligence because I had my hidden identities. And now… I've had so many experiences and stuff, I trust myself a bit more. And I think that comes with age.

The relationship between confidence and identity varied widely person to person and could be challenging to predict.

Other benefits of certain identities seemed more consistent such as those that affected the participants' ability to attend the course. For example, one participant talked about how attending graduate school in a particular country gave them access to financial resources that they would not have had at home,

From my home institution, I've received quite a lot of financial support. I've received some grants from institutions and also applied for another grant, so that's really helpful. But again, I don't think that would be possible if I'm not in [country]. Because, just to give you an example, the course fee is actually like three months' salary in my home country. So it's… I wouldn't save my three months' salary just for this course, you know?

Relatedly, one participant acknowledged that their higher socioeconomic status granted them benefits that either *decrease anxiety or stress, or increase comfort* while attending the course,

I don't struggle to be like, worrying about staying in a place in [City] the night before or the night after or like getting transport here, or if we want to go to the pub and we want to sit and have a chill drink… I don't have to worry about that. So it takes a level of stress away.. to know that I don't have to worry about financial stuff.

This participant references benefits related to their socioeconomic status including getting to the course, ease of lodging before or after the course, and ways they might choose to relax or find entertainment before, during or after the course.

A different participant perceived that their educational background may have aided in getting them accepted into the course even if that did not align with course admission policies,

First of all, the background that I have allowed me to get into the course. Because I mean, they were quite clear about how competitive it was and that there were a lot of good applicants. So I think my [advanced educational] background helped [me] to get in.

Both of these participants saw their identities as playing a key role in even being able to attend the course in the first place, thereby acknowledging the assets that can come with these identities. Furthermore, despite explicit need‐based selection criteria for this particular course (see above) they still perceived that having an advanced educational background aided participants both in getting into the field course and with being successful once they were in attendance. One participant shared that their pre‐existing content knowledge not only made the course easier but also *promoted* their *academic engagement*. The participant said,

We had lectures before the course to get a baseline, but I feel like I already had a lot of background in those topics, and so it was easier for me to engage and have discussions and stuff.

Specifically, already having this content knowledge made it easier for this participant to engage in group discussions and apply their knowledge to the topic of the field course.

Identities could also benefit individuals in regard to actively participating and finding their role as part of the course community. Several participants referenced their identities as helping *improve their communication and connection* with others in the course. In some cases, participants found that sharing identities with their peers, such as being a scientist, made conversations easier and more exciting. One participant described this in this way,

Being here and surrounded by people who have those shared interests and really love to talk about those types of ideas and that type of science has been a really great opportunity. I can finally… have those types of conversations and have them be really interesting and fun…I feel like my educational background has definitely allowed me to do that because I feel like I've learned a lot and finally have people who want to share in that knowledge.

For this individual, the field course provided new opportunities to connect with people in a way that they didn't usually. This shared passion made the participant excited to engage in the course and converse with their peers. Sharing identities, for example, as scientists or as individuals interested in natural history, with a significant number of people in the course was regularly seen as advantageous.

For students who were from a shared cultural background, this shared identity could make connection easier. One reflected on their experience saying,

Often we want to find the other people of similar backgrounds. And if you can quickly, in the first couple of days of the course, like, okay, I'm trying to be friends with everyone, I'm trying to get along with everyone, but there's a few people I know that I can have a certain conversation with that I wouldn't be able to with other people.

Another participant shared how being from a country that was overrepresented at the course helped them engage in conversations and potentially even participate in course activities,

I feel like there is an outsized number of [people from one country] on this course and being from [that country], at least a good amount of the conversation has been around, you know, kind of [that country]‐centric topics at times. And so, I guess that makes it easier to socialize and maybe also to participate. I guess… Several of the course leaders are from [that country] as well, so we might have, you know, more common ground to start upon.

This participant notes that sharing a country of origin with both peers and course leaders benefitted them because they were able to contribute to common topics of discussion beyond the course content.

Finally, one participant recognized that their identity gave them a practical advantage,

I feel like I can express myself when anything's going wrong and I have the whole vocabulary and then what I say will be perceived correctly because most people speak English here.

Speaking English as a native language helped this participant circumvent or address problems as they came up in the course. Being able to express oneself can have a myriad of benefits both practically and socially.

Overall, it was not simply one type of identity that could benefit students in the course. Many different identities could help with course preparation, discussions, and social interactions depending on the point of view and potentially the intersectionality of the individual. In particular, when a participant held an identity that was commonly represented among course attendees, that participant was able to note the advantages that identity gave them.

### 3.4 Social Promoters for Engagement

Given that many of the potential barriers to engagement focused on or involved social interactions with peers or communications with course leaders, having supportive leaders and peers in this particular field course helped many participants engage. Prompt communication, “commitment,” and “respect” were used by participants to describe positive interactions with course leaders. These factors could be expressed as supportive actions or sentiments from their peers and from the course leaders. This *support from course leaders* could be both practical and emotional. A few participants described times where a course leader went out of their way to provide additional aid to them, such as one course leader who,

realized we were just left behind and looking very miserable…and [they] stepped in, like [they] dropped everything in terms of probably four hours out of [their] day, maybe more, to hike us up to the site and make sure we knew [where] we were going…I felt like [they] demonstrated that [they] really cared about us and our group.

In this example, the participant saw the course leader as going above and beyond to make sure that the students were successful in their responsibilities.

*Open‐minded peers* were also valued by participants, especially when reflecting upon what made them comfortable to engage in the course while holding a CSI or nonconcealable stigmatized identity. Some participants described this open‐mindedness as a general sense of inclusion or welcome. For example, “I had the impression that everyone [*said with emphasis]* was very welcoming so I felt like, at the meals, like I felt like I could sit anywhere and it wasn't a problem.” Other participants referred to specific conversations with or aid that they received from their peers. One participant stated,

[One of the peers], she gave me a map and like, I made a point on the map and then explained the way, so I could find the way alone. And that helped, that I could decide by myself how quickly [to hike], when do I need to stop, and so on.

In this moment, the peer was able to help the participant navigate their own abilities and access needs by empowering them with information. Yet another participant described how their peers' acceptance of them being a non‐native English speaker made it easier for them to navigate social interactions,

I remember the other day I had a conversation with someone in my group and then I was saying ‘I feel like I'm kind of stupid because I just don't know how to respond [to peers]. I don't really know how to let the conversation flow,’ even if that's not true…I had those bad experiences in the past that makes me think that I'm not good enough, or kind of imposter syndrome and that keeps happening until now and my peers, like other students, have been supportive for that. They appreciate my effort. When I try to like, say something, they've been very supportive about that.

Given the social pressures and expectations that come with field courses, having peers who create safer environments in which individuals can navigate these social discourses was very important for some participants to engage in the course.

Support from both course leaders and peers has *reduced fieldwork difficulties* for the student. One example can be seen in the quote above where simply sharing where the end point of a trip was empowered a participant to set their own pace and take breaks as they needed. Another example was when peers or course leaders shared equipment, clothing, or knowledge. One participant described this as “looking out for one another” and specifically stated that this attitude “mitigated” the barrier of their inexperience with the climate at the field sites.

### 3.5 Participating in the Interview Prompted Reflection

A few participants remarked upon how participating in the interview for this study actually helped them process their experiences. Others appeared to reflect on the relationship between varying identities and the course more deeply while talking with the interviewer than they may have otherwise. One participant commented directly on the impact of having this study occur during the course,

I'm sure that they've done their best, but sometimes like people just… if people don't have experience of being underrepresented, they just couldn't relate…So yeah, I feel like this kind of… having this kind of conversation or having this kind of study within this course is something that would be helpful … for the future courses.

This participant saw the ongoing study and the interviews as ways to communicate and as a signal that the course leadership cared about issues of diversity and equity. However, participants also pointed out issues that they had come across in the course.

Many participants noted that the diversity of the students in the course was fairly limited and that this *lack of diversity* was sometimes present in the course materials as well. One participant critiqued the course sharing,

Just having White people in the slides of any material being presented is problematic. There should just be representation that there's just not like White people or White women doing science, there's people all over the world. And the people conducting this work look differently than what has been represented and shown thus far.

The lack of diversity in the course materials was one concern, but another was the make‐up of the course participants. A participant even extrapolated the demographics of the course participants to their view of population of the discipline as a whole saying, “I feel like I had this really skewed view of ecology as being like a field that's like generally quite good with diversity and then in that course, I was like ‘wow [*with emphasis*], it's not.’” Lacking diversity among the students could potentially impact individuals' experiences with the course and during the interviews a few participants with majority identities reflected on that. They noted that the lack of diversity among course participants might make some of their peers uncomfortable such as one White participant who said,

I think there were, I don't even know, like three people of color on the course. I don't even have a real number…And there might have been more, but it was 35‐something people who were all White. And that's a course with a lot of like homogeneity… I imagine others might have felt, like the other people of color, might have been very aware of this very White‐dominated course.

A couple of participants took this line of thinking one step further, noting that field courses have the potential to be a “high impact teaching practice” but to maximize that potential impact the people that need the course most have to be able to attend. A participant explained,

To have high impact, it has to target those [who need] the course the most. But I don't think that's what has been done by the organizers. So mostly it's like those who can pay this [course fee], who might have similar interests, or this kind of thing. It's not like those who need the course the most.

Note that this perception persisted within this course despite the need‐based criteria, variable and waivable course fees, and blinded selection process, which were communicated to the students beforehand (see above).

Students who did not have marginalized identities were also able to reflect during the interviews on how the structure, funding setup, and demographic makeup of field courses could all contribute to feelings of isolation and lessen the positive outcomes of these types of courses for some students.

### 3.6 Suggestions for Inclusive Field Courses Provided by Study Participants

While reporting students' suggestions for how to improve field courses was not an original aim of the project, students still provided ideas for course development, often in relation to barriers they had personally experienced in one course or another. Here, we summarize the suggestions as they were provided by the study participants; however, we do not include recommendations that were highly specific to the structure or content of this particular course as they would not be broadly applicable to others. There were four core areas for improvement mentioned by the participants: (1) admissions, (2) structuring social opportunities, (3) establishing course culture, and (4) addressing the needs of individual students.

First, participants acknowledged that having a diverse student population is a direct route to creating a space where more students felt represented and comfortable. They recognized that course costs, while not in the power of the instructors, can be a major barrier. The participants further suggested that an equity and access‐minded admissions process focused on diversifying the student group could significantly enhance field course outcomes. Furthermore, these processes would need to be designed to address the fact that some students have privileges that afford them access to research experiences and other resources that would make them more competitive while other students may appear less competitive simply because they had been denied other opportunities. For example, in the course participants were recruited from, the admission process was “blinded” to avoid unconscious biases during selection, and the selection criteria focused on the academic needs and added value of the course for the students, rather than their prior experience (see above). When applied together, these aspects help reduce the potential impact of both unconscious bias and prior privileges in the selection process.

Second, approximately half of the participants wanted to see more course structures that encouraged socializing and working with students who were not in their assigned groups. Many field courses, including the one sampled from in this study, are centered around group projects where small groups of students are collectively responsible for carrying out and reporting on their project. Often, these groups will be operating in different parts of a field site or prioritizing different tasks, ultimately causing them to spend little time together across groups. Because of this, many participants recommended more integration between groups. Some suggested that this could be done by switching up the groups, having groups swap members for a day, or pairing different groups to work together.

However, switching groups of students during a field course can be logistically infeasible, hinder the students' mastery of their own groups' projects, and could also make it difficult to develop good collaborative dynamics within the groups. With this in mind, many participants suggested that creating more shared activities that required students to talk to each other across the course could have similar benefits to mixing up the groups. Essentially, any opportunities to shuffle students around and create dedicated time to talk to those not in their groups were welcomed by the participants. They proposed that this would not only have social benefits, but also encourage more learning as students would get opportunities to learn from more peers and course leaders than those in their immediate group.

Third, there were two different suggestions that focused around messaging and course climate. Participants noted that there could be significant communication about the value of diversity and about equitable treatment of each other in course materials and communications, including in the meetings before a course begins and in the materials advertising for a course. They also recommended a “soft landing” for students upon arriving at any field site. In detail, the participants suggested dedicating more time to setting up group culture, developing group expectations for behavior, and getting to know their peers. One participant did acknowledge that the pre‐course virtual meetings for this particular course were helpful to feel like others in the course were not complete strangers when arriving, but that some additional time to get comfortable with each other in person would have also helped them feel more at ease.

Finally, many of the participants mentioned that course leaders should attend to the needs of individual students. Some had felt lost in the sea of students and hoped for some way for course leaders to track or check‐in on each student throughout the course. Participants commented that some students might fall ill or need a piece of equipment and wanted clearly defined mechanisms to express those needs to course leaders. For this suggestion, not only does there need to be a mechanism to check‐in on students, but also the resources to follow‐up with those students and address their needs.

## 4 Discussion

We describe findings from a series of interviews intended to understand how student salient identities, representing multiple axes of diversity, served to either promote or hinder engagement in an international, graduate‐level field course. We drew inspiration from many colleagues who have described specific challenges students face in field courses (e.g., Treibergs et al. 2022; Lemmons 2015; Cronin et al. 2024). For example, Treibergs et al. (2022) take a broad view of student challenges (e.g., scientific, logistic) but does not specifically probe the interaction between engagement and student identities. Cronin et al. (2024) address the need to mitigate the problem of sexual harassment at field sites, and Lemmons (2015) focuses on student learning while embedded in a new culture. The work described here is the first—to our knowledge—to delve into identity salience this broadly, with consideration for both concealable and less concealable identities, and their impacts on field course engagement.

First, we return to our research questions and summarize our findings in response.

1. Which identities were salient to students during this international, graduate‐level field course?

Students volunteered a variety of different identities as salient during their course participation. These ranged in how concealable they were, whether they were typically seen as stigmatized, and whether they were minorities in this particular course setting. In this work, we document six broad themes relating to identity and inclusion in graduate‐level, international field courses. These themes are: *Identity as a barrier, Social barriers associated with one's identity, Identity as a promoter, Supportive peers and instructors*, and *Interview as intervention*. The first five themes broadly reflect the interplay between student contextual factors (e.g., their identities, worldviews, prior experiences) and field experience design factors (e.g., social interactions during the course, orientation and experience within the course), as laid out in the Undergraduate Field Experiences Research Network (UFERN) model (O'Connell et al. 2022). The UFERN model posits that both student contextual factors and course design factors impact each student's experience, and thus impact the resulting student outcomes.

2 Did identity salience serve to promote or serve as barriers to student engagement in this course? specifically, in what ways did identity salience promote or serve as a barrier to student engagement in this course?

Students noted both barriers and promoters associated with their salient identities, with some noting that a single identity could present as either a barrier or a promoter to engagement, depending on context. We were especially intrigued by the students who noted an incoming concern about an identity being a barrier, and then finding during the course that these concerns were unfounded. We clearly see this realization as positive, but we are also aware that just the incoming concern and awareness of a potential vulnerability is a type of psychological threat (specifically, “identity threat”; Branscombe et al. 1999, Steele et al. 2002) that can exert a cost on an individual. For example, a student may ultimately realize their identity is not problematized by their peers or teachers, but prior to that realization they may experience stress or fear of alienation—emotions that detract cognitive resources from course activities and may impede their social integration into the course.

3 How can we use insight from these interviews to design courses that promote inclusion for participants from a range of backgrounds and with diverse identities?

Several respondents provided suggestions for mitigating barriers and enhancing promoters to engagement. We summarize and expand upon many of those suggestions in the following section.

### 4.1 Implications for Improving Field Courses

**FIGURE 2:**
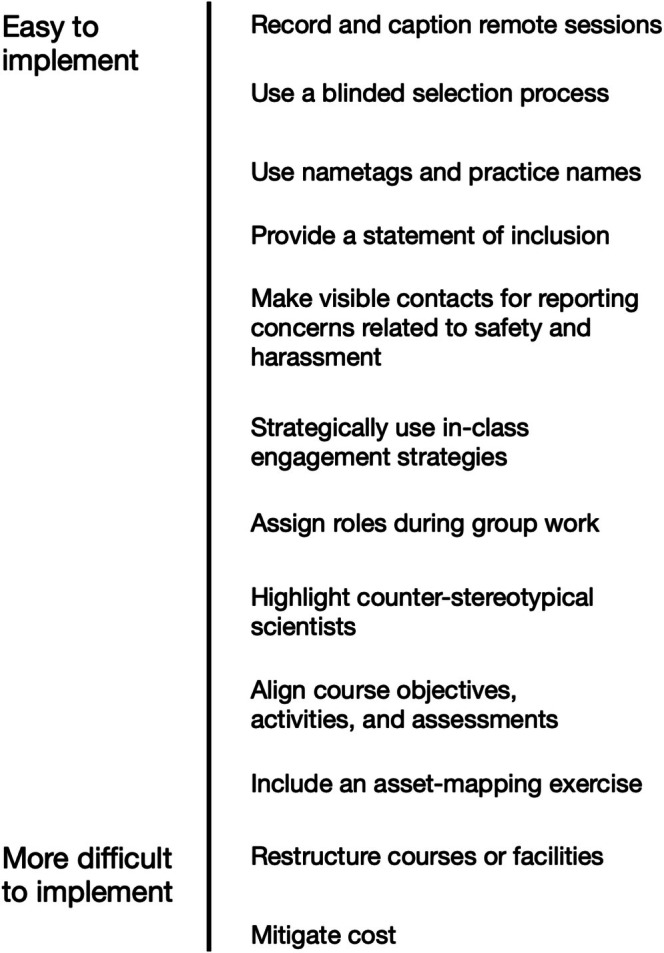
Overview of our recommendations, from those most easy to implement to those that require more effort.

Here, we shift focus to the final theme *suggestions for improvement* that are focused on suggestions for developing field courses that support students in mobilizing their diverse identities as assets for their learning process, while minimizing factors that result in identities becoming barriers. We draw on student suggestions and topics identified in the interviews, existing literature, and an Asset‐Based Pedagogy framing to suggest how to promote diversity and inclusion in field courses. In general, we encourage making field courses more inclusive through incorporating ABP whenever possible, and we provide suggestions below. Notably, most of the suggestions were directly from or inspired by participant suggestions. We also acknowledge that stepping into inclusive field teaching for the first time can feel overwhelming, thus we include considerations of effort in our recommendations. Specifically, our suggestions range from those that are the easiest to implement to those requiring a more fundamental reallocation of content or resources (summarized in Figure 2).

The course in this study used remote pre‐course lectures and group discussions as an onboarding strategy to familiarize students with the course topic and background literature, the topic and tasks of each group, study areas, and the leaders and other participants. Recordings of the lectures and discussions were provided on the course home page to ensure access and opportunity to revisit. Such onboarding discussions can help mitigate incoming concerns about expectations and peers, but additional considerations could be beneficial. Captioning lectures and discussions would allow everybody—including those needing more time due to language barriers, connectivity challenges, or neurodivergence—to process the information.

A group of student comments suggested students had not fully understood or did not remember some features of the course–the explicitly blinded and need‐based student selection process, opportunities to borrow necessary personal equipment, variable and waivable course fees, etc. This suggests that very explicit communication, repetition, and online visibility of course policies, including communication of the motivation behind and outcomes of such policies, may be critical in supporting students' perceptions of a wider range of their salient identities as assets and promoters, rather than barriers. Secondly, several of our findings suggested that dominance of the student body by one nationality, culture, or language group can negatively impact group dynamics and become a perceived barrier for students who do not belong to that group, while promoting the dominant (and, in our case, already self‐identified as ‘privileged’) group. More explicit policies aiming to achieve a balanced student population across many diversity dimensions, going beyond the traditional focus on, for example, gender and ethnicity, to also consider for example, language, culture, socioeconomic background, technical capabilities, and familiarity with outdoors work, could benefit students in many ways. We also suggest that simple strategies like using name tags on the first day of in‐person meetings can promote a sense of belonging, especially if students and teachers use that as an opportunity to learn and use names (Cooper et al. 2017).

The course in this study has a course philosophy readily available at the course web pages that clarifies the scientific, educational, collegial, and outreach philosophy of the course. Such simple statements of inclusion in the course syllabus or on the website can communicate to students that you value equity and inclusion and that you recognize some students may encounter barriers that others do not face (Fuentes et al. 2021). This statement can be strengthened with descriptions of the specific actions you plan to take to promote inclusion, as well as contact information for reporting any concerns related to belonging in the course. Similarly, personal safety and harassment contacts should be visible in course syllabi or related resources, and they should include individuals outside of those leading the course. These strategies can be impactful and require little effort or course time.

We also suggest that simple strategies like using name tags on the first day of in‐person meetings can promote a sense of belonging, especially if students and teachers use that as an opportunity to learn and use names (Cooper et al. 2017). Instructors can also implement a variety of in‐class engagement strategies that are designed to encourage participation from all students (we recommend Tanner 2013 as a starting place), several of which can be used both in the classroom and field settings. For example, a “think‐pair‐share” approach when posing thoughtful questions to students encourages all students–even the most outgoing–to pause and think, to share in the comfort of a small group and give feedback to their peers, and then to participate in whole‐group discussion (Kaddoura 2013; Kothiyal et al. 2013). Further, several investigators have highlighted the benefits of assigning roles during group work, among them equity and representation benefits (Smith 1996); the same logic can be applied to group work in a field‐course setting. This also ensures that everyone gets hands‐on experience with field‐work specific tasks such as planning and organizing equipment, safety and operations during field work, operating equipment and making measurements, and taking notes and recording progress. Further, this ensures that everyone in the group has a chance to speak and voice their opinions on decisions made and changes of plans during the field campaign.

In designing examples, and in selecting literature, instructors can make an effort to include work from a diverse group of scientists, and also highlight counter‐stereotypical scientists. Field‐based research can be great settings for these kinds of approaches, highlighting local scientists and their work for an international group of students. These efforts will not only provide role models for students from underrepresented identities in STEM, but they will also communicate to students your commitment to inclusion. Good resources for using role models in STEM courses include Scientist Spotlights (https://scientistspotlights.org/, Schinske et al. 2016; Yonas et al. 2020) and Project Biodiversify (https://projectbiodiversify.org/, Zemenick et al. 2022).

Instructors must also realize they are modeling what a scientist is. They can model open and inclusive communication, including approaches that allow everyone to engage, set clear expectations and boundaries, demonstrate respect for diversity, collaborate with local scientists, prioritize safety and well‐being during field work, and share experiences and equipment for how to stay warm, energized, and comfortable during field work, including in challenging weather and physical situations. Several students in this course reflected on broadly shared interests and values among students and instructors—in this course around science, natural history, and environmental concerns. Such shared interests and values could be explicitly leveraged as opportunities for connection, dialogue, and onboarding around topics like what it means to be a scientist and the role of science and scientists in society.

Instructors can also take care to align course objectives, activities, and assessments to reduce the ‘hidden curriculum’ that can be a barrier to many first‐generation and non‐majority culture students. If a course goal is to learn, for example, a collection technique in the field, then training and assessment can focus on how to achieve the entire process, from planning via the conduction in the field to data management and reporting, in a reproducible, safe, and comfortable manner, going beyond the technical step of operating equipment. If the goal is to learn how to do good science, then embedding the various learning activities in ‘real’ research gives many opportunities for learning, while promoting career‐enhancing outcomes. In this course, for example, the final student product was a published data paper, co‐produced by students and instructors (Vandvik et al. 2025). This gives a “real” experience of the entire research process, including many steps that especially first‐generation students may be less familiar with.

Because many students shared that the interviews we conducted served as an intervention that in itself promotes inclusivity, we have considered how a similarly reflective activity could be incorporated into the curriculum for field courses in general. Instructors could, for example, borrow from the Asset‐Based Pedagogy literature and use an asset‐mapping exercise in the beginning of a field course (Borrero and Sanchez 2017). Among the diverse assets students might possess are those related to “hand, head, and heart” domains (Rippon and Hopkins 2015; Gazibara 2013). In the context of field courses, physical assets (“hand”) can involve outdoor work experience, orientation, first aid, craft, or mechanical skills; intellectual assets (“head”) can include speaking multiple languages, taking careful notes, a strong theoretical background, or having in‐depth knowledge of the flora or natural history of a particular region; and interpersonal assets (“heart”) can include overcoming challenges, handling inclement weather, navigating conflicts, and being especially conscious of the welfare of others. While there are several suggestions for asset‐mapping activities elsewhere (e.g., https://www.cswe.org/CSWE/media/Diversity‐Center/5‐Exercises.pdf), the key feature of these activities is that they recognize students as complex individuals and highlight diverse aspects of their identities. These features often go unrecognized and under‐appreciated, but they can be important to accomplishing our goals in the multifaceted environment of a practical or field course (Sipos et al. 2008).

Pairing asset‐mapping with assigning group roles could help students feel like they are taking on roles and responsibilities that they are best prepared for, potentially empowering students to take ownership and agency in the course activities. Depending on the course assignments and goals, there could be non‐traditional roles in a field course that allow students to tap into a variety of skills and strengths. For example, field courses that involve working with local communities and scientists could benefit from students who could serve as translators or who see themselves as skilled in science communication, teaching, or negotiation. In past iterations of the focal course for this work, students have engaged in a “science communication module” in which they surveyed local residents (near the field sites) about their knowledge and perspectives of climate change (Patrick et al. 2020). Students conducted this work, in local communities, in groups, drawing on diverse interests and skills within the groups–fluency in the native language (e.g., Spanish in Peru, Norwegian on Svalbard), facility with conversing with strangers, organizing notes in the field, etc. Other assets that could be leveraged in the field include skills with navigation, using maps, providing first aid, managing inventory, labeling and organizing data, carrying or moving equipment, making sure their peers all get to participate, or numerous other tasks that go beyond simple content knowledge.

Considerably more effort is required to restructure courses or facilities. For our colleagues developing courses de novo, we recommend involving instructors that represent multiple axes of diversity and considering whether alternative activities, to accommodate those with different abilities, would meet your course goals. If facilities are planned for development, we urge consideration of unnecessarily gendered spaces, and where possible, ramps instead of–or in addition to–stairs. In the course we described, students felt they had a student representative (i.e., the interviewer) with whom they could share concerns to be communicated to course leaders. Many students, both interview participants and nonparticipants, pointed out that just having the representative there as a go‐between was helpful. This led to a suggestion that *all* field courses have a person whose basic responsibilities concern student and instructor well‐being (including diversity, equity and inclusion, safety, and sexual harassment). One important role of this individual could be anonymously communicating student questions or concerns to all course instructors, or even training as a coach or mediator to aid in personal or interpersonal stresses.

Because social interactions between students are more prevalent within a field course than a traditional classroom, purposefully building community as part of the formal course activities could aid in students' sense of belonging (Race et al. 2021). Strategic activities where the entire class or subgroups of students and instructors introduce themselves, discuss their goals for the course, and establish norms of how they wish to communicate to each other could aid students with hidden identities. Figuring out social norms of communication can be challenging for everyone, and establishing and renegotiating these norms transparently and throughout a collaboration can aid in group function (Mullins 2018). These conversations could provide space to demystify the hidden curriculum of field courses and incorporating faculty into these conversations could help build rapport between students and instructors; however, it will be important to let students' lead when establishing norms due to the power dynamic between instructors and students. Community building is another area where students could bring in their underappreciated assets; individuals with less privilege are likely to be more aware of the hidden curriculum or notice unclear expectations. Those individuals could help guide these conversations to make sure expectations are demystified for everyone.

Related to both asset‐mapping, modeling what a scientist is, and building community comes the idea of highlighting students' shared identities as both scientists and students. While the students of any given course will vary widely in their personal identities, they do all share identities as burgeoning scientists and as students. Furthermore, being part of a group of scientists was one of the ‘promoting’ salient identities recognized in our interviews. Research has repeatedly shown that having a strong identity as a scientist can help students be successful in their courses (Osborne and Walker 2006; Chen et al. 2021), weather moments of failure, and persist in science (Hernandez et al. 2017). We propose that encouraging students to bond over their shared identities as scientists could also aid in building community between peers by focusing on their shared goals and interests.

Among the largest barriers to engagement in field courses, and one that was repeatedly mentioned in our interviews, is cost, and that is a barrier unlikely to be solved by the strategies suggested above. Field experiences can be included in the formal curriculum (thus folding the expenses into the costs of education; Haigh and Gold 1993; Peacock et al. 2018). International courses, like the one studied here, offer added values of building an international network and learning in ‘real’ research settings beyond one's own university. Many universities, learned societies, and national and international research educational councils or programs offer economic support for student field work and course participation, and information about such options can help more students get access to them. Organizers can mitigate costs by supporting field courses through external funding, varying course fees by countries, and offering opportunities for a need‐based waiving fees. Course developers can also consider working with institutions to provide low‐cost dormitory‐style housing options, and by, when possible, seeking field sites that are accessible by public transportation. But honestly, the extreme differences in wealth and privilege that create barriers to field course engagement create barriers in almost all aspects of society. We speculate, however, that simply recognizing financial disparities as a barrier to engagement is a critical first step. Any efforts we take to counter these disparities and promote access to these valuable experiences will likely result in improvements.

### 4.2 Considerations and Limitations

We must note that our ability to extrapolate to similar field courses has limitations. Field course curricula are highly variable, and this course is an established and tested curriculum that has resulted in positive student evaluations, publications, and the formation of lasting networks. The course is also inter‐institutional and externally funded, and has hosted 180 participants from 86 institutions and 30 countries over its 7 iterations, posing a set of unique opportunities but also limitations. Based on past experiences and feedback, the course now includes: precourse seminars and meetings to build community and provide time to read up and prepare beforehand; detailed packing lists and also equipment to borrow; a “blinded” student selection process that prioritize students according to their academic needs (and not merits); an emphasis on regional students to build networks; subsidized course fees due to external funding with variable fee rates depending on the country of the student's academic institution; and fee adjustment for certain students. While other courses are likely to face some different challenges and opportunities than those documented here, participants made a number of comments that may be of use for developing this and other field courses.

Furthermore, we are limited–by ethical considerations–from referring to specific features of a student's identity. As a result, our conclusions and recommendations are general, a restriction that may make our implications more broadly applicable while failing to address specific issues that can arise with respect to specific identities.

Partway through the research project, the researchers also noted that the resulting data had significantly greater focus on barriers students experienced in relation to their identities rather than strengths that they brought to the field course. We believe that there are multiple potential reasons for this focus on barriers. First, while the original interview questions incorporated both barriers and benefits, follow‐up questions during the interviews were often focused on clarifying and understanding the barriers. Second, barriers may be easier to reflect upon for participants as barriers are often more immediate than benefits, the latter of which may also require more in‐depth reflection to recognize. Therefore, the results do not fully align with asset‐based pedagogy, which emphasizes the benefits and strengths that diverse individuals bring with them into a space. However, we report on both the strengths and the barriers students reported experiencing and provide recommendations on how to incorporate ABP more fully in the future.

## 5 Conclusion

We do not pretend to understand all the challenges students face in field courses, nor are all these challenges related to student identities. However, with this investigation we disclose some identity‐based barriers and promoters to engagement in field courses, along with recommendations for countering some of these barriers and maximizing the promoters. Given the extensive benefits to students provided by participating in field courses, we assert that any unnecessary barriers should be recognized and addressed. We call on our colleagues to consider which of these barriers may be relevant in their own teaching context and how they might work to make field courses more equitable and inclusive.

## Author Contributions

**Alyssa N. Olson:** conceptualization (equal), formal analysis (lead), methodology (lead), writing – original draft (equal), writing – review and editing (equal). **Sehoya Cotner:** conceptualization (equal), methodology (supporting), writing – original draft (equal), writing – review and editing (equal). **Vigdis Vandvik:** conceptualization (supporting), funding acquisition (lead), methodology (supporting), writing – review and editing (equal). **A. Kelly Lane:** conceptualization (supporting), formal analysis (equal), methodology (equal), writing – original draft (lead), writing – review and editing (equal).

## Funding

This work was supported by a grant from the Norwegian Research Council awarded to Vigdis Vandvik (#274831).

## Conflicts of Interest

The authors declare no conflicts of interest.

## Data Availability

The datasets referenced in this article are not readily available because the approved study protocol and consent form follow GDPR and thus explicitly state that data will be anonymized and not shared with external parties. Requests for information about the interview transcripts should be directed to the corresponding author Sehoya Cotner (sehoya.cotner@uib.no).

## Endnotes

^1^
In this manuscript, we elect to capitalize White when in reference to a racial identity as recommended in 2020 by the National Association of Black Journalists in the United States. This choice aligns with the perspective that capitalizing White challenges the perception that Whiteness is equivalent to racial neutrality or absence. However, we do acknowledge that there is ongoing debate on this topic (e.g., Appiah 2020; NABJ 2020; Painter 2020; Coleman 2020; Meir 2020).

## References

[ece373230-bib-0001] Appiah, K. A. 2020. “The Case for Capitalizing the “B” in Black.” The Atlantic. https://www.theatlantic.com/ideas/archive/2020/06/time‐to‐capitalize‐blackand‐white/613159/.

[ece373230-bib-0002] Arboleya, A. M. , and B. González‐Díaz . 2021. “Intercultural Experiential Learning: Integrated Geography Field Courses for Undergraduates in Arts and Humanities in Spain.” In Experiential Learning in Geography, edited by J. E. Wessell . Springer. 10.1007/978-3-030-82087-9_10.

[ece373230-bib-0003] Arcila Hernández, L. M. , A. Borker , R. S. Beltran , A. I. Race , and E. S. Zavaleta . 2024. “Fostering Inclusion in the Life Sciences Through Course‐Based Field Research Experiences.” Assessment Update 36, no. 5: 12–13. 10.1002/au.30413.

[ece373230-bib-0004] Arcila Hernández, L. M. , C. S. Mittan‐Moreau , T. Lamb , et al. 2023. “A Half Century of Student Data Reveals the Professional Impacts of a Biology Field Course.” Bioscience 73, no. 1: 59–67.

[ece373230-bib-0005] Beltran, R. S. , E. Marnocha , A. Race , D. A. Croll , G. H. Dayton , and E. S. Zavaleta . 2020. “Field Courses Narrow Demographic Achievement Gaps in Ecology and Evolutionary Biology.” Ecology and Evolution 10, no. 12: 5184–5196. 32607142 10.1002/ece3.6300 PMC7319162

[ece373230-bib-0006] Booth, A. , C. Carroll , I. Ilott , L. L. Low , and K. Cooper . 2013. “Desperately Seeking Dissonance: Identifying the Disconfirming Case in Qualitative Evidence Synthesis.” Qualitative Health Research 23, no. 1: 126–141. 23166156 10.1177/1049732312466295

[ece373230-bib-0007] Borrero, N. , and G. Sanchez . 2017. “Enacting Culturally Relevant Pedagogy: Asset Mapping in Urban Classrooms.” Teaching Education 28, no. 3: 279–295.

[ece373230-bib-0008] Branscombe, N. R. , N. Ellemers , R. Spears , and B. Doosje . 1999. “The Context and Content of Social Identity Threat.” In Social Identity: Context, Commitment, Content, edited by N. Ellemers , R. Spears , and B. Doosje , 35–58. Blackwell.

[ece373230-bib-0009] Braun, V. , and V. Clarke . 2006. “Using Thematic Analysis in Psychology.” Qualitative Research in Psychology 3, no. 2: 77–101.

[ece373230-bib-0010] Braun, V. , V. Clarke , N. Hayfield , and G. Terry . 2018. “Thematic Analysis.” In Handbook of Research Methods in Health Social Sciences, edited by P. Liamputtong . Springer. 10.1007/978-981-10-2779-6_103-1.

[ece373230-bib-0011] Braun, V. , V. Clarke , and N. Rance . 2014. “How to Use Thematic Analysis With Interview Data.” In The Counselling & Psychotherapy Research Handbook, edited by A. Vossler and N. Moller , 183–197. Sage.

[ece373230-bib-0012] Brendel, N. , F. Aksit , S. Aksit , and G. Schrüfer . 2016. “Multicultural Group Work on Field Excursions to Promote Student Teachers' Intercultural Competence.” Journal of Geography in Higher Education 40, no. 2: 284–301.

[ece373230-bib-0013] Carlone, H. B. , and A. Johnson . 2007. “Understanding the Science Experiences of Successful Women of Color: Science Identity as an Analytic Lens.” Journal of Research in Science Teaching 44, no. 8: 1187–1218. 10.1002/tea.20237.

[ece373230-bib-0014] Casper, A. A. , N. Rebolledo , A. K. Lane , L. Jude , and S. L. Eddy . 2022. “’It's Completely Erasure’: A Qualitative Exploration of Experiences of Transgender, Nonbinary, Gender Nonconforming, and Questioning Students in Biology Courses.” CBE Life Sciences Education 21, no. 4: ar69. 36112619 10.1187/cbe.21-12-0343 PMC9727607

[ece373230-bib-0015] Chen, S. , K. R. Binning , K. J. Manke , et al. 2021. “Am I a Science Person? A Strong Science Identity Bolsters Minority Students' Sense of Belonging and Performance in College.” Personality and Social Psychology Bulletin 47, no. 4: 593–606. 32659167 10.1177/0146167220936480 PMC7961640

[ece373230-bib-0016] Clancy, K. B. , R. G. Nelson , J. N. Rutherford , and K. Hinde . 2014. “Survey of Academic Field Experiences (SAFE): Trainees Report Harassment and Assault.” PLoS One 9, no. 7: e102172 Cleveland Clinic. (2022, June). Neurodivergent. https://my.clevelandclinic.org/health/symptoms/23154‐neurodivergent. 25028932 10.1371/journal.pone.0102172 PMC4100871

[ece373230-bib-0017] Coleman, N. 2020. Why We're Capitalizing Black. New York Times. https://www.nytimes.com/2020/07/05/insider/capitalized‐black.html.

[ece373230-bib-0018] Cooper, K. M. , and S. E. Brownell . 2016. “Coming Out in Class: Challenges and Benefits of Active Learning in a Biology Classroom for LGBTQIA Students.” CBE Life Sciences Education 15, no. 3: ar37. 27543636 10.1187/cbe.16-01-0074 PMC5008884

[ece373230-bib-0019] Cooper, K. M. , V. R. Downing , and S. E. Brownell . 2018. “The Influence of Active Learning Practices on Student Anxiety in Large‐Enrollment College Science Classrooms.” International Journal of STEM Education 5: 1–18. 30631713 10.1186/s40594-018-0123-6 PMC6310416

[ece373230-bib-0020] Cooper, K. M. , L. E. Gin , and S. E. Brownell . 2020. “Depression as a Concealable Stigmatized Identity: What Influences Whether Students Conceal or Reveal Their Depression in Undergraduate Research Experiences?” International Journal of STEM Education 7: 1–18. 10.1186/s40594-020-00216-5 PMC7271012 32550126

[ece373230-bib-0021] Cooper, K. M. , B. Haney , A. Krieg , and S. E. Brownell . 2017. “What's in a Name? The Importance of Students Perceiving That an Instructor Knows Their Names in a High‐Enrollment Biology Classroom.” CBE Life Sciences Education 16, no. 1: ar8. 28188281 10.1187/cbe.16-08-0265 PMC5332051

[ece373230-bib-0022] Creswell, J. W. , and D. L. Miller . 2000. “Determining Validity in Qualitative Inquiry.” Theory Into Practice 39, no. 3: 124–130.

[ece373230-bib-0023] Cronin, M. R. , E. S. Zavaleta , R. S. Beltran , et al. 2024. “Testing the Effectiveness of Interactive Training on Sexual Harassment and Assault in Field Science.” Scientific Reports 14, no. 1: 523. 38191560 10.1038/s41598-023-49203-0 PMC10774269

[ece373230-bib-0024] D'Amico, M. M. , and S. L. Dika . 2013. “Using Data Known at the Time of Admission to Predict First‐Generation College Student Success.” Journal of College Student Retention: Research, Theory & Practice 15, no. 2: 173–192.

[ece373230-bib-0025] Downing, V. R. , K. M. Cooper , J. M. Cala , L. E. Gin , and S. E. Brownell . 2020. “Fear of Negative Evaluation and Student Anxiety in Community College Active‐Learning Science Courses.” CBE Life Sciences Education 19, no. 2: ar2. 32453679 10.1187/cbe.19-09-0186 PMC8697658

[ece373230-bib-0026] Dudley‐Marling, C. 2015. “The Resilience of Deficit Thinking.” Journal of Teaching and Learning 10, no. 1: 1–12.

[ece373230-bib-0027] Durrant, K. L. , and T. P. V. Hartman . 2015. “The Integrative Learning Value of Field Courses.” Journal of Biological Education 49, no. 4: 385–400. 10.1080/00219266.2014.967276.

[ece373230-bib-0028] Dwyer, S. C. , and J. L. Buckle . 2009. “The Space Between: On Being an Insider‐Outsider in Qualitative Research.” International Journal of Qualitative Methods 8, no. 1: 54–63.

[ece373230-bib-0029] Esparza, D. , and M. K. Smith . 2023. “Professional Social Connections Are Associated With Student Science Identity in a Research‐Based Field Biology Course.” Ecosphere 14, no. 9: e4662.

[ece373230-bib-0030] Fägerstam, E. 2014. “High School Teachers' Experience of the Educational Potential of Outdoor Teaching and Learning.” Journal of Adventure Education & Outdoor Learning 14, no. 1: 56–81.

[ece373230-bib-0031] Fuentes, M. A. , D. G. Zelaya , and J. W. Madsen . 2021. “Rethinking the Course Syllabus: Considerations for Promoting Equity, Diversity, and Inclusion.” Teaching of Psychology 48, no. 1: 69–79.

[ece373230-bib-0032] Gazibara, S. 2013. “‘Head, Heart and Hands Learning’‐A Challenge for Contemporary Education.” Journal of Education, Culture, and Society 4, no. 1: 71–82.

[ece373230-bib-0033] Geange, S. R. , J. Von Oppen , T. Strydom , et al. 2021. “Next‐Generation Field Courses: Integrating Open Science and Online Learning.” Ecology and Evolution 11, no. 8: 3577–3587. 33898010 10.1002/ece3.7009 PMC8057340

[ece373230-bib-0034] Giles, S. , C. Jackson , and N. Stephen . 2020. “Barriers to Fieldwork in Undergraduate Geoscience Degrees.” Nature Reviews Earth & Environment 1, no. 2: 77–78.

[ece373230-bib-0035] Haigh, M. , and J. R. Gold . 1993. “The Problems With Fieldwork: A Group‐Based Approach Towards Integrating Fieldwork Into the Undergraduate Geography Curriculum.” Journal of Geography in Higher Education 17, no. 1: 21–32.

[ece373230-bib-0036] Hall, T. , M. Healey , and M. Harrison . 2004. “Fieldwork and Disabled Students: Discourses of Exclusion and Inclusion.” Journal of Geography in Higher Education 28, no. 2: 255–280. 10.1080/0309826042000242495.

[ece373230-bib-0037] Henn, J. J. , B. J. Enquist , A. H. Halbritter , et al. 2018. “Intraspecific Trait Variation and Phenotypic Plasticity Mediate Alpine Plant Species.” Frontiers ‐ Topics in Plant Science 9: 1548. 10.3389/fpls.2018.01548. PMC6243391 30483276

[ece373230-bib-0038] Henning, J. A. , C. J. Ballen , S. A. Molina , and S. Cotner . 2019. “Hidden Identities Shape Student Perceptions of Active Learning Environments.” Frontiers in Education 4: 129.

[ece373230-bib-0039] Hernandez, P. R. , B. Bloodhart , R. T. Barnes , et al. 2017. “Promoting Professional Identity, Motivation, and Persistence: Benefits of an Informal Mentoring Program for Female Undergraduate Students.” PLoS One 12, no. 11: e0187531. 29091969 10.1371/journal.pone.0187531 PMC5665547

[ece373230-bib-0040] Hotez, E. , K. A. Rosenau , P. Fernandes , et al. 2022. “A National Cross‐Sectional Study of the Characteristics, Strengths, and Challenges of College Students With Attention Deficit Hyperactivity Disorder.” Cureus 14, no. 1: e21520. 35223296 10.7759/cureus.21520 PMC8865363

[ece373230-bib-0041] Hoyer, L. , and W. W. Hastie . 2023. “Geoscience Undergraduate Students' Perceptions of How Field Work and Practical Skills Influence Their Conceptual Understanding and Subject Interest.” Journal of Geoscience Education 71, no. 2: 158–176. 10.1080/10899995.2022.2110630.

[ece373230-bib-0042] Hughes, B. E. 2018. “Coming Out in STEM: Factors Affecting Retention of Sexual Minority STEM Students.” Science Advances 4, no. 3: eaao6373. 29546240 10.1126/sciadv.aao6373 PMC5851677

[ece373230-bib-0043] Jensen, A. J. , S. P. Bombaci , L. C. Gigliotti , et al. 2021. “Attracting Diverse Students to Field Experiences Requires Adequate Pay, Flexibility, and Inclusion.” Bioscience 71, no. 7: 757–770. 34220360 10.1093/biosci/biab039 PMC8245294

[ece373230-bib-0044] Kaddoura, M. 2013. “Think Pair Share: A Teaching Learning Strategy to Enhance Students' Critical Thinking.” Educational Research Quarterly 36, no. 4: 3–24.

[ece373230-bib-0045] Kothiyal, A. , R. Majumdar , S. Murthy , and S. Iyer . 2013. “Effect of Think‐Pair‐Share in a Large CS1 Class: 83% Sustained Engagement.” In Proceedings of the Ninth Annual International ACM Conference on International Computing Education Research (pp. 137–144).

[ece373230-bib-0046] Kuh, G. D. 2008. “Excerpt From High‐Impact Educational Practices: What They Are, Who Has Access to Them, and Why They Matter.” Association of American Colleges and Universities 14, no. 3: 28–29.

[ece373230-bib-0047] Kuh, G. D. , and K. O'Donnell . 2013. Ensuring Quality & Taking High‐Impact Practices to Scale. Association of American Colleges & Universities.

[ece373230-bib-0048] Lemmons, K. 2015. “Short‐Term Study Abroad: Culture and the Path of Least Resistance.” Journal of Geography in Higher Education 39: 543–553. 10.1080/03098265.2015.1084607.

[ece373230-bib-0049] López, F. 2024. “Asset‐Based Pedagogy.” In Handbook of Educational Psychology, 433–457. Routledge.

[ece373230-bib-0050] Lundin, M. , and S. Bombaci . 2023. “Making Outdoor Field Experiences More Inclusive for the LGBTQ+ Community.” Ecological Applications 33, no. 5: e2771. 36271764 10.1002/eap.2771

[ece373230-bib-0051] Maia, M. C. , G. Lamego , C. I. Elliff , J. M. Del Favero , J. Leonel , and C. R. Marcolin . 2024. “Harassment and Bullying Aboard: Impacts of Gender Inequality on Ocean Professionals.” Marine Policy 160: 105946.

[ece373230-bib-0052] Marín‐Spiotta, E. , R. T. Barnes , A. A. Berhe , et al. 2020. “Hostile Climates Are Barriers to Diversifying the Geosciences.” Advances in Geosciences 53: 117–127.

[ece373230-bib-0053] McGrath, C. , P. J. Palmgren , and M. Liljedahl . 2019. “Twelve Tips for Conducting Qualitative Research Interviews.” Medical Teacher 41, no. 9: 1002–1006. 10.1080/0142159X.2018.1497149. 30261797

[ece373230-bib-0054] Meir, N. 2020. Why We Will Lowercase White. Associated Press. https://www.ap.org/the‐definitive‐source/announcements/why‐we‐will‐lowercase‐white/.

[ece373230-bib-0055] Morales, N. , K. Bisbee O'Connell , S. McNulty , et al. 2020. “Promoting Inclusion in Ecological Field Experiences: Examining and Overcoming Barriers to a Professional Rite of Passage.” Bulletin of the Ecological Society of America 101, no. 4: e01742.

[ece373230-bib-0056] Mulholland, J. 2007. “Understanding the Self as Instrument.” In Contemporary Qualitative Research: Exemplars for Science and Mathematics Educators, edited by P. C. Taylor and J. Wallace , 45–57. Springer Netherlands. 10.1007/978-1-4020-5920-9_5.

[ece373230-bib-0057] Mullins, S. B. 2018. “Establishing a Community of Discourse Through Social Norms.” Discourse and Communication for Sustainable Education 9, no. 1: 5–17.

[ece373230-bib-0058] NABJ . 2020. NABJ Statement on Capitalizing Black and Other Racial Identifiers. National Association of Black Journalists. https://nabjonline.org/blog/nabj‐statement‐on‐capitalizing‐black‐and‐other‐racial‐identifiers/.

[ece373230-bib-0059] Nash, M. , H. E. F. Nielsen , J. Shaw , M. King , M.‐A. Lea , and N. Bax . 2019. “‘Antarctica Just Has This Hero Factor…’: Gendered Barriers to Australian Antarctic Research and Remote Fieldwork.” PLoS One 14, no. 1: e0209983. 10.1371/journal.pone.0209983. 30650104 PMC6334902

[ece373230-bib-0060] Niu, L. 2017. “Family Socioeconomic Status and Choice of STEM Major in College: An Analysis of a National Sample.” College Student Journal 51, no. 2: 298–312.

[ece373230-bib-0061] Núñez, A.‐M. , J. Rivera , and T. Hallmark . 2020. “Applying an Intersectionality Lens to Expand Equity in the Geosciences.” Journal of Geoscience Education 68, no. 2: 97–114. 10.1080/10899995.2019.1675131.

[ece373230-bib-0062] O'Connell, K. , K. L. Hoke , M. Giamellaro , A. R. Berkowitz , and J. Branchaw . 2022. “A Tool for Designing and Studying Student‐Centered Undergraduate Field Experiences: The UFERN Model.” Bioscience 72, no. 2: 189–200. 10.1093/biosci/biab112.

[ece373230-bib-0064] Osborne, J. W. , and C. Walker . 2006. “Stereotype Threat, Identification With Academics, and Withdrawal From School: Why the Most Successful Students of Colour Might Be Most Likely to Withdraw.” Educational Psychology 26, no. 4: 563–577.

[ece373230-bib-0065] Painter, N. I. 2020. Opinion Why ‘White’ Should be Capitalized, Too. Washington Post. https://www.washingtonpost.com/opinions/2020/07/22/why‐white‐should‐be‐capitalized/.

[ece373230-bib-0066] Parson, L. 2019. “Considering Positionality: The Ethics of Conducting Research With Marginalized Groups.” In Research Methods for Social Justice and Equity in Education, edited by K. K. Strunk and L. A. Locke , 15–32. Springer International Publishing. 10.1007/978-3-030-05900-2_2.

[ece373230-bib-0067] Patrick, L. , S. Thompson , A. H. Halbritter , B. J. Enquist , V. Vandvik , and S. Cotner . 2020. “Adding Value to a Field‐Based Course With a Science Communication Module on Local Perceptions of Climate Change.” Bulletin of the Ecological Society of America 101, no. 3: e01680.

[ece373230-bib-0068] Peacock, J. , R. Mewis , and D. Rooney . 2018. “The Use of Campus Based Field Teaching to Provide an Authentic Experience to All Students.” Journal of Geography in Higher Education 42, no. 4: 531–539.

[ece373230-bib-0069] Peasland, E. L. , D. C. Henri , L. J. Morrell , and G. W. Scott . 2019. “The Influence of Fieldwork Design on Student Perceptions of Skills Development During Field Courses.” International Journal of Science Education 41, no. 17: 2369–2388.

[ece373230-bib-0070] Pierszalowski, S. , J. Bouwma‐Gearhart , and L. Marlow . 2021. “A Systematic Review of Barriers to Accessing Undergraduate Research for STEM Students: Problematizing Under‐Researched Factors for Students of Color.” Social Sciences 10, no. 9: 328.

[ece373230-bib-0071] Quinn, D. M. 2006. “Concealable Versus Conspicuous Stigmatized Identities.” In Stigma and Group Inequality, 97–118. Psychology Press.

[ece373230-bib-0072] Race, A. I. , R. S. Beltran , and E. S. Zavaleta . 2021. “How an Early, Inclusive Field Course Can Build Persistence in Ecology and Evolutionary Biology.” Integrative and Comparative Biology 61, no. 3: 957–968. 10.1093/icb/icab121. 34089317 PMC8490690

[ece373230-bib-0073] Rippon, S. , and T. Hopkins . 2015. Head, Hands and Heart: Asset‐Based Approaches in Health Care. Health Foundation.

[ece373230-bib-0074] Roos, R. E. , J. Kemppinen , P. Niittynen , et al. 2025. “Marine‐Derived Nutrients Shape the Functional Composition of High Arctic Plant Communities.” Functional Ecology 39, no. 6: 1606–1621.

[ece373230-bib-0075] Rudzki, E. N. , and K. D. Kohl . 2023. “Deficits in Accessibility Across Field Research Stations for Scientists With Disabilities and/or Chronic Illness, and Proposed Solutions.” Integrative and Comparative Biology 63, no. 1: 114–127. 37156525 10.1093/icb/icad019

[ece373230-bib-0076] Saldaña, J. 2016. The Coding Manual for Qualitative Researchers. 3rd ed. SAGE.

[ece373230-bib-0078] Schinske, J. N. , H. Perkins , A. Snyder , and M. Wyer . 2016. “Scientist Spotlight Homework Assignments Shift Students' Stereotypes of Scientists and Enhance Science Identity in a Diverse Introductory Science Class.” CBE Life Sciences Education 15, no. 3: ar47. 27587856 10.1187/cbe.16-01-0002 PMC5008894

[ece373230-bib-0079] Shaulskiy, S. , A. Jolley , and K. O'Connell . 2022. “Understanding the Benefits of Residential Field Courses: The Importance of Class Learning Goal Orientation and Class Belonging.” CBE Life Sciences Education 21, no. 3: ar40. 35763331 10.1187/cbe.21-08-0201 PMC9582821

[ece373230-bib-0080] Shinbrot, X. A. , K. Treibergs , L. M. A. Hernández , et al. 2022. “The Impact of Field Courses on Undergraduate Knowledge, Affect, Behavior, and Skills: A Scoping Review.” Bioscience 72, no. 10: 1007–1017. 10.1093/biosci/biac070. 36196223 PMC9525126

[ece373230-bib-0081] Siedlecki, S. L. 2022. “Conducting Interviews for Qualitative Research Studies.” Clinical Nurse Specialist 36, no. 2: 78–80. 10.1097/NUR.0000000000000653.

[ece373230-bib-0082] Sipos, Y. , B. Battisti , and K. Grimm . 2008. “Achieving Transformative Sustainability Learning: Engaging Head, Hands and Heart.” International Journal of Sustainability in Higher Education 9, no. 1: 68–86.

[ece373230-bib-0083] Smith, K. A. 1996. “Cooperative Learning: Making’ Groupwork’ Work.” New Directions for Teaching and Learning 1996: 71–82.

[ece373230-bib-0084] Steele, C. M. , S. J. Spencer , and J. Aronson . 2002. “Contending With Group Image: The Psychology of Stereotype and Social Identity Threat.” In Advances in Experimental Social Psychology, vol. 34, 379–440. Academic Press.

[ece373230-bib-0085] Strayhorn, T. L. 2018. College Students' Sense of Belonging: A Key to Educational Success for All Students. Routledge.

[ece373230-bib-0086] Tanner, K. D. 2013. “Structure Matters: Twenty‐One Teaching Strategies to Promote Student Engagement and Cultivate Classroom Equity.” CBE Life Sciences Education 12, no. 3: 322–331. 24006379 10.1187/cbe.13-06-0115 PMC3762997

[ece373230-bib-0087] Thompson, S. K. , S. Hebert , S. Berk , et al. 2020. “A Call for Data‐Driven Networks to Address Equity in the Context of Undergraduate Biology.” CBE Life Sciences Education 19, no. 4: mr2. 10.1187/cbe.20-05-0085. 33001771 PMC8693933

[ece373230-bib-0088] Thomson, E. R. , M. Spiegel , I. H. J. Althuizen , et al. 2021. “Multiscale Mapping of Plant Functional Groups and Plant Traits in the High Arctic Using Field Spectroscopy, UAV Imagery and Sentinel‐2A Data.” Environmental Research Letters 16: 055006. 10.1088/1748-9326/abf464.

[ece373230-bib-0089] Treibergs, K. A. , D. Esparza , J. A. Yamazaki , M. Goebel , and M. K. Smith . 2022. “How Do Introductory Field Biology Students Feel? Journal Reflections Provide Insight Into Student Affect.” Ecology and Evolution 12, no. 11: e9454. 36407897 10.1002/ece3.9454 PMC9666715

[ece373230-bib-0090] Vandvik, V. , A. H. Halbritter , M. Macias‐Fauria , et al. 2025. “Plant Traits and Associated Ecological Data From Global Change Experiments and Climate Gradients in Norway.” Scientific Data 12, no. 1: 1477. 10.1038/s41597-025-05509-4. 40854906 PMC12379105

[ece373230-bib-0091] Wang, K. D. , J. McCool , and C. Wieman . 2024. “Exploring the Learning Experiences of Neurodivergent College Students in STEM Courses.” Journal of Research in Special Educational Needs 24: 505–518.

[ece373230-bib-0092] Yonas, A. , M. Sleeth , and S. Cotner . 2020. “In a “Scientist Spotlight” Intervention, Diverse Student Identities Matter.” Journal of Microbiology & Biology Education 21, no. 1: 10–1128. 10.1128/jmbe.v21i1.2013 PMC7148145 32313593

[ece373230-bib-0093] Zavaleta, E. S. , R. S. Beltran , and A. L. Borker . 2020. “How Field Courses Propel Inclusion and Collective Excellence.” Trends in Ecology & Evolution 35, no. 11: 953–956. 10.1016/j.tree.2020.08.005. 32919797 PMC7480643

[ece373230-bib-0094] Zemenick, A. T. , S. C. Jones , A. J. Webster , et al. 2022. “Diversifying and Humanizing Scientist Role Models Through Interviews and Constructing Slide Decks on Researchers' Research and Life Experiences.” CourseSource 9: 1–13.

